# A Pilot Survey of Career and Professional Advising of Medical Schools in the United States

**DOI:** 10.15694/mep.2021.000141.1

**Published:** 2021-05-23

**Authors:** Lisa Shah-Patel, Eric vanSonnenberg, Paul Kang, Susan Kaib

**Affiliations:** 1 University of Arizona College of Medicine-Phoenix; 2 University of Arizona Mel and Enid Zuckerman College of Public Health

**Keywords:** Advising, undergraduate medical education, professionalism, US medical schools, career advising

## Abstract

This article was migrated. The article was marked as recommended.

**Purpose:** Scant information is available about the makeup of Career and Professional Advising systems, and who the advisors are in U.S. medical schools. We created a survey in 2019 and collated the responses to gain information about Advisors and advising systems.

**Materials and Methods:** An 11 question survey was emailed to 72 U.S. medical schools, querying information about whether they had a Career and Professional Advising system and what is the construct of the system. Kruskal Wallis and Fisher’s Exact tests were utilized for analysis.

**Results:** 30/72 responses were received (41.67%). Educational backgrounds of advisors included: 27/30 (90%) by physicians; 7/30 (23.3%) by PhDs; 9/30 (30%) by Masters; 4/30 (13.3%) by others. AAMC Careers in Medicine curriculum was delivered in 23/30 (75.7%). Most advising systems were in Student Affairs (27/30, 90%), although only 20/30 (66.7%) reported to the Dean of Student Affairs.

**Conclusion:** There was no unanimity in any of the responses to the 11 questions about who Career and Professional Advisors are, or how the systems are constructed. The closest to unanimity is that most medical schools have advising systems, that they are in Student Affairs departments, and that most advisors are physicians.

## Introduction

Career and Professional Advising has become an essential aspect of medical student support in U.S. medical schools. Advisors function to help students with career planning, professionalism, core competencies, academic issues, residency applications, research and volunteer opportunities, medical specialty preparation, and a host of other *ad hoc* issues. Qualifications for Career and Professional advising are not codified nor uniform. Some institutions utilize non-physician advisors such as those with a Master’s degree or PhD to help with career planning, while others use non-specialty specific physician advisors. Other institutions utilize these aforementioned types of advisors for years 1 and 2 of medical school, then pair their students with a specialty specific physician advisor for clerkship and rotations in years 3 and 4.

Overall, Career and Professional Advising is not a standardized, streamlined system amongst U.S. medical schools; anecdotally, there is quite a bit of variability. Thus, we devised a survey to help clarify and characterize medical school Career and Professional advising and advisors. Given the variability of educational backgrounds and experience of advisors, and that there are no specific standards for advising systems within U.S. medical schools, we sought to obtain information about both Career and Professional Advising and about the advisors themselves through a pilot survey. This information, besides for shedding light, might potentially guide advising for new medical schools, and help standardize and optimize Career and Professional Advising.

## Methods

Seventy-two U.S. medical schools were contacted by emails that were available to Deans of Students (n=42) and to attendees (n=30) of a recent AAMC course in Washington, D.C. on Career and Professional Advising. Email addresses were obtained through the AAMC office and through the Associate Dean of Student Affairs at our medical school. The emails and responses were collated over a five month span.

An 11 question online email survey was prepared through Qualtrics. The survey queried whether or not the medical school has a specific advising system, background information about the advisors, the total number of FTEs, volunteer versus paid advisors, AAMC advising curriculum implementation, reporting structure, and specialty versus general advisors. All responses were anonymous. The entire survey is in Appendix 1. IRB was waived by the IRB Committee. This work was conducted following current ethical guidelines and was approved by University of Arizona Research, Discovery, and Innovation.

Statistical assessment was performed using Kruskal Wallis for continuous comparison, and Fisher’s Exact for categorical variables.

## Results/Analysis

Thirty responses (of 72) were received (41.67%). 93.3% (28/30) of institutions have a dedicated advising system. Educational backgrounds of advisors were the following: 27/30 (90%) by physicians; 7/30 (23.3%) by PhDs; 9/30 (30%) by Masters; 4/30 (13.3%) by others. The median FTE at the schools for advising was 1, although class sizes varied from 75-180. The median number of paid advisors was 2; the median number of volunteer advisors was 15. Paid versus volunteer advising was equal at 26.7% (8/30); a combination was reported in 14/30 (46.7%). The AAMC Careers in Medicine curriculum was delivered in 23/30 (75.7%) institutions.

27/30 (90%) of advisors were in Student Affairs Departments; 5/30 (16.7%) were in Academic Affairs; 17/30 (56.7%) were in clinical specialties; 2/30 (6.67%) were “other”. 7/30 (23.3%) of advisors were specialty specific. Advisor reporting was as follows: to the Dean of Student Affairs 20/30 (66.7%), to the Director of Advising 3/30 (10%), to Academic Affairs 2/30 (6.67%), to the Dean of the medical school 1/30 (3.3%), and “other” in 3/30 (10%).

## Discussion

Not many specifics are known about who performs medical school Career and Professional Advising, who the advisors are and their backgrounds, and how advising systems are constructed in U.S. medical schools. Anecdotally, some schools maintain an open advising system, in which students can seek out an advisor/mentor in their specialty area of interest; other schools assign a general advisor to each student from the start of medical school throughout all four years, while others have a hybrid system, in which students are part of a specific advisor group or society for years 1 and 2, then obtain a specialty specific advisor for years 3 and 4. Our goal in this study was to gain information about who the advisors are, their backgrounds, and how institutions set up their advising programs. This knowledge should provide insight into the spectrum of Career and Professional Advising in U.S. medical schools, and could be useful for schools that are implementing advising programs.

Our data show that although over 90% of institutions surveyed have a dedicated advising system, the organization of these advising systems and the makeup and background of the advisors vary widely. The responses suggest that there is no standardization of Career and Professional Advising in the responding medical schools. Marked variability in educational backgrounds, reporting structures, volunteer versus paid, host departments, and general versus specific advisors characterized the responses.

The AAMC Careers in Medicine curriculum is delivered in ¾ of the institutions, but how and who delivers this important curricular information varies. The motivations and availability of advisors, along with individual institutional specifics, likely influence these variations. The closest metrics to unanimity (although none was) were the presence of an advising system, physicians utilized as the advisors, and that Student Affairs departments housed the advisors.

The literature is scant with respect to the medical school advising organizational questions in our Advisor survey. An interesting approach to advising was documented by the Weill Cornell Medical College. They revamped a previously unsatisfactory advising program by having the Associate Dean for Student Affairs and the program director to specifically select 50 highly-qualified faculty members to advise one to three medical students in the students’ first two years ( Drusin *et al.,* 2013). Some students, did not meet with their originally assigned advisors, and found their own faculty members as advisors. These, and other authors ( Wilson, 2004; Rose *et al.,* 2005; Mellman and Paquette, 2012; Aagaard, 2015; Howse *et al.,* 2017), described a spectrum of different approaches to advising, but they concluded that, “a single clear pathway to success has not emerged.” A multi-institutional study found themes from advisors that influenced medical students to pursue family medicine as a career ( Alavi *et al.,* 2019). An article from Columbia University College of Physicians and Surgeons noted that advising programs varied in names, scope, and structures; hence, they implemented a formal Advisory Dean Program that provided personalized mentoring and advising for each student ( Macauley *et al.,* 2007). while these papers and programs do not specifically address the exact questions we posed in our survey, nonetheless, similar to our study, they document the heterogeneity of medical student advising.

The main limitation to our study is the small sample size. The survey was sent to emails that were available to us through our Dean of Student Affairs, and via a AAMC workshop list of course attendees. Hence, the study must be considered a pilot, as only about ½ of the medical schools in the US were queried, and slightly less than ½ responded.

## Conclusion

In conclusion, despite the limitations, both similarities and notable differences were highlighted in the survey. The information should be of interest to medical schools with Career and Professional Advising programs, as well as for schools that are forging nascent programs. Hopefully, this pilot information can help stimulate conversation and further studies to elucidate and optimize Career and Professional medical student advising.

## Take Home Messages

• Information on Career and Professional Advising systems in U.S. Medical Schools is scant
• No unanimity is seen in who the Advisors are and how the systems are constructed
• Most medical schools though have advising systems in Student Affairs and most advisors are physicians

## Notes On Contributors

**Lisa Shah-Patel** is the Interim Associate Dean of Student Affairs, Director of Career and Professional Advising and a Clinical Associate Professor in the Department of Radiology at the University of Arizona College of Medicine-Phoenix.

**Eric vanSonnenberg** is a Career and Professional Advisor and Professor in the Department of Radiology at the University of Arizona College of Medicine-Phoenix.

**Paul Kang** is a Biostatistician at the University of Arizona College of Public Health-Phoenix.

**Susan Kaib** is a Career and Professional Advisor and a Clinical Associate Professor in the Department of Family, Community, and Preventive Medicine at the University Arizona College of Medicine-Phoenix.

## Appendices

### Appendix 1: Advisor Survey

Thank you for agreeing to complete the survey. It should take no more than 5 minutes, as the mechanisms to respond are constructed in a simple and straightforward format. We will share the information to all of you once it’s collated anonymously. Thank you very much.

1. Institution:

2. How many students does your institution matriculate per year?

3. Does your institution have a dedicated career advising system for medical students? (yes or no)

4. What is the total FTE for the advisors (e.g. FTE of 1 = 40 h\week, FTE of 0.5 = 20hrs/week; if your Medical School has 5 advisors who are all full-time, total FTE is 5)?

5. Are your advisors...? (Paid, Volunteers, or we have paid and Volunteer Advisors)

6. How many advisors are paid and how many are volunteers?

Paid Advisors

Volunteer Advisors

7. Do you have a dedicated advising system to deliver the Careers in Medicine curriculum per AAMC? (yes, no, or N/A)

8. Who does the Career and Professional advising at your institution? (Choose all that apply)

MD

PhD

Masters

Other

9. If you have Advisors, in what medical school department or clinical department are they in? (Choose all that apply)

Student Affairs

Academic Affairs

Clinical Specialty

Other

10. Do your advisors only advise those students in their own specialties? (e.g. If the advisor is a general surgeon, does he/she only interact with those students planning on applying to general surgery?)

(yes, no, or N/A)

11. Whom do the advisors report to?

Dean of Students

Director of Advising
