# Supplementary material for: A Pilot Survey of Career and Professional Advising of Medical Schools in the United States
Source: MedEdPublish (2016). 2021 May 23;10:141. [Version 1] doi: 10.15694/mep.2021.000141.1 (PMC10939549; doi:10.15694/mep.2021.000141.1)
Supplement: Peer review history

# Reviewer report: Reviewer response for version 1

**DOI:** 10.21956/mep.18926.r27059

**Published:** 2021-07-13

**Authors:** Gert Van Zyl

**Affiliations:** 1 University of the Free State

This review has been migrated. The reviewer awarded 4 stars out of 5 Excellent article that addresses an area to take note of. Enjoyed reading it. Wondered if the response rate was better, if the outcome and findings would have stayed the same. The heterogeneity of medical student advising as found is a challenge. It may be worthwhile to follow up on students' view and what they perceive to be the best advising and to focus on a model build on that to take student advising forward!

Reviewer Expertise:

NA

No decision status is available

# Reviewer report: Reviewer response for version 1

**DOI:** 10.21956/mep.18926.r27060

**Published:** 2021-07-13

**Authors:** Richard Hays

**Affiliations:** 1 James Cook University

This review has been migrated. The reviewer awarded 4 stars out of 5 I found this article interesting and highly relevant to accreditation standards for medical education. All sets of standards that I know of list student support systems as mandatory, although precise structures and functions are neither dictated nor prescribed. Accreditation panels make judgements based on how participants feel that their support systems works. The idea of exploring these issues is sound and the methods are appropriate. The findings are not really surprising and are mostly reassuring - most programs have what appear to be at least reasonable arrangements. Follow up is required to investigate how well their support system works. For example, what does 'reporting to Associate Deans' mean? This could be a voice of advocacy in higher places (a plus) but could also mean that student support services get caught up in progress decisions (a minus), depending on the precise roles of the Associate Deans. The views of students are also important, as it may be possible to identify structures and functions that are more beneficial for students. Overall a worthwhile read for all involved in student support systems.

Reviewer Expertise:

NA

No decision status is available
